# The importance of using video marketing in ophthalmology

**DOI:** 10.22336/rjo.2022.21

**Published:** 2022

**Authors:** Consuela-Mădălina Gheorghe

**Affiliations:** * Philologist, Authorized translator

Video marketing is, according to the mass-media literature, a component of the online marketing. It is important to mention that consumers will more likely purchase ophthalmology services or products after watching an online video. Compared to the other means of promotion and advertising, such as blogs, social media or websites, videos do not require expensive equipment, therefore, the costs to produce them are low. 

Videos can be used to explain examinations, meaning what patients should expect when going to the ophthalmologist (private clinic), because they may be reluctant to even going to a simple consultation, let alone an ophthalmological surgery. Moreover, they can be used to promote services, gain new patients or patient testimonials. The aim of video marketing is to gain the patients’ trust and to help them remember the ophthalmological clinic, services and products, accounting for over 50% of all traffic registered from mobile devices and being preferred over reading a text. 

Educational videos can also be shared with the purpose of raising awareness regarding an ophthalmological procedure or the steps to be followed during a consultation. 

Video marketing has major benefits for the ophthalmology clinic, such as the fact that the information is easily shared if people like the video they see, the clinic may easily be considered an expert in the field of ophthalmology, as the video offers the ability to present the product(s), service(s), or proficiency. Thus, when you are seen, people tend to believe more in all you have to offer.

A “behind the scenes” video is arguably one of the most useful videos an ophthalmology clinic can use. Although these videos are not quite as polished and scripted as practice advertising ones, they are nevertheless useful resources for giving people more information about the clinic.

Behind-the-scenes videos are frequently used to provide brief “glimpses” into the routine of an ophthalmology clinic on social media. These kinds of videos should be used to give patients a chance to learn more about the clinic and the staff who works there.

A video has a powerful potential that few other types of communication do in addition to trying to educate patients: it can humanize. Putting a face on something that is not naturally personified, like an ophthalmology clinic, might help potential patients “identify” with the business and feel more at ease before they even go to the clinic.

Patients will only entrust their vision to someone they believe they can trust because human faces usually foster connection, sincerity, and trust.

The general rules of videos are that they should provide value, meaning that they should offer solutions to the patients’ problems, they should be remembered, meaning that they should contain a script that can be easily be remembered visually by the patients, they should be remarkable, meaning that they should be catchy and not bore the patient, they should be short, meaning that they should not exceed 30 seconds and they should concentrate on the visual effects rather than the words, and they should be posted everywhere, meaning that they should be seen on all social media sites. 

Finally, yet importantly, videos can also contain patients’ testimonials, this being a tool for attracting more potential patients.

In conclusion, video marketing can help an ophthalmology clinic become more visible and strengthen its brand identity by forging a much more personal relationship with the patients. Moreover, video marketing is not new, and it has grown to be a potent and popular marketing tool.

